# Interference with Amyloid-β Nucleation by Transient Ligand Interaction

**DOI:** 10.3390/molecules24112129

**Published:** 2019-06-05

**Authors:** Tao Zhang, Jennifer Loschwitz, Birgit Strodel, Luitgard Nagel-Steger, Dieter Willbold

**Affiliations:** 1 Institute of Complex Systems, Structural Biochemistry (ICS-6), Forschungszentrum Jülich, 52425 Jülich, Germany; tao.zhang@uni-duesseldorf.de (T.Z.); j.loschwitz@fz-juelich.de (J.L.); b.strodel@fz-juelich.de (B.S.); l.nagel-steger@fz-juelich.de (L.N.-S.); 2 Institut für Physikalische Biologie, Heinrich-Heine-Universität Düsseldorf, 40225 Düsseldorf, Germany; 3 Institute of Theoretical and Computational Chemistry, Heinrich-Heine-Universität Düsseldorf, 40225 Düsseldorf, Germany

**Keywords:** amyloid-β peptides, aggregation, complex formation, D-enantiomeric peptide, intrinsically disordered protein

## Abstract

Amyloid-β peptide (Aβ) is an intrinsically disordered protein (IDP) associated with Alzheimer’s disease. The structural flexibility and aggregation propensity of Aβ pose major challenges for elucidating the interaction between Aβ monomers and ligands. All-D-peptides consisting solely of D-enantiomeric amino acid residues are interesting drug candidates that combine high binding specificity with high metabolic stability. Here we characterized the interaction between the 12-residue all-D-peptide D3 and Aβ42 monomers, and how the interaction influences Aβ42 aggregation. We demonstrate for the first time that D3 binds to Aβ42 monomers with submicromolar affinities. These two highly unstructured molecules are able to form complexes with 1:1 and other stoichiometries. Further, D3 at substoichiometric concentrations effectively slows down the β-sheet formation and Aβ42 fibrillation by modulating the nucleation process. The study provides new insights into the molecular mechanism of how D3 affects Aβ assemblies and contributes to our knowledge on the interaction between two IDPs.

## 1. Introduction

Intrinsically disordered proteins (IDPs) are a group of proteins which lack stable secondary and tertiary structures [1]. The structural flexibility of these proteins is often relevant for their functional roles in various biological activities [2]. In addition to physiological functions, IDPs are also implicated in protein misfolding diseases, in which the misfolding and abnormal aggregation of one or more IDPs are considered to be crucial early events in disease pathogenesis [3]. Amyloid-β peptide (Aβ) is one of the key molecules in the pathogenesis of Alzheimer’s disease (AD) [4]. Aβ is composed of 39 to 43 amino acid residues and cleaved from the amyloid precursor protein (APP) [5,6]. Aβ monomers in aqueous environments have been recognized as IDPs due to their overall random coil structures [7]. However, Aβ monomers are able to form ordered conformations upon self-assembling into toxic oligomers, which are the most relevant species for disease development and progression [8], as well as into fibrillar structures [9]. The central role of Aβ in the pathogenesis of AD has raised a lot of interest in identifying physiological or non-physiological molecules that can modulate the aggregation process of Aβ and antagonize toxic Aβ oligomers.

Based on the rationale that stabilization of Aβ in its monomeric IDP conformation should efficiently inhibit the aggregation of Aβ, and even destabilize and ultimately eliminate already existing toxic Aβ assemblies, we carried out a mirror image phage display selection, and obtained “D3”, a peptide consisting of 12 D-enantiomeric amino acid residues [10,11]. D-enantiomeric peptides comprise a promising substance class for the development of therapeutic agents because of their high potency and low proteolytic susceptibility [12]. The arginine-rich D3 peptide lacks well-defined structural features, and can thus be described as a small IDP. D3 demonstrated its beneficial effects in targeting Aβ species in vitro and in AD transgenic mouse models [13], as well as its stability [14,15], while the detailed mechanism of action underlying the interaction between the highly flexible D3 and Aβ monomers remained elusive. Specific high-affinity interactions between intrinsically disordered proteins are something, which has not been expected until very recently [16]. The inherent flexibility of the binding partners, and the dynamic nature of the interaction, contradict the existence of a well-defined 1:1 complex structure, as one would expect in the case of a typical receptor-ligand-complex, which is principally accessible to high-resolution structural biology methods. This excludes the existence of a well-defined three-dimensional structure of ‘the’ complex that would be amenable to routine high resolution structure determination methods. The strong aggregation propensity of Aβ poses additional challenges to distinguish ligand binding from self-association. Due to the structural diversity and opposite charges of D3 and Aβ monomers, we reasoned that the interaction between these two molecules is not a standard ligand-receptor interaction. Instead, it might resemble a specific high affinity interaction between two small IDPs, characterized by a variable stoichiometry.

To characterize the interaction between D3 and Aβ42 monomers, which is essential for understanding the mechanism of the action of D-peptides on Aβ pathology, we applied solution-based fluorescence approaches to investigate the binding and complex formation between D3 and monomeric Aβ42 at concentrations as low as possible to avoid confounding aggregation-related artifacts, wherever feasible. Experimental data on the interaction from fluorescence-based analytical ultracentrifugation (AUC-FDS) and microscale thermophoresis (MST) was supported by molecular dynamics (MD) simulations. Furthermore, the influence of D3 on the aggregation of Aβ42 was studied at clearly substoichiometric ratios by employing a thioflavin T (ThT) assay, circular dichroism (CD) spectroscopy and atomic force microscopy (AFM). By combining various experimental techniques and MD simulations, we set out to clarify the interaction between unstructured D3 and Aβ42 monomers. The findings will not only deepen our knowledge on the mechanism of action of D3, but also shed some light on the formation of IDP-IDP complexes in general.

## 2. Results

### 2.1. Characterization of the Dissociation Constant of Aβ42 and D3 Interaction

**Figure 1:**
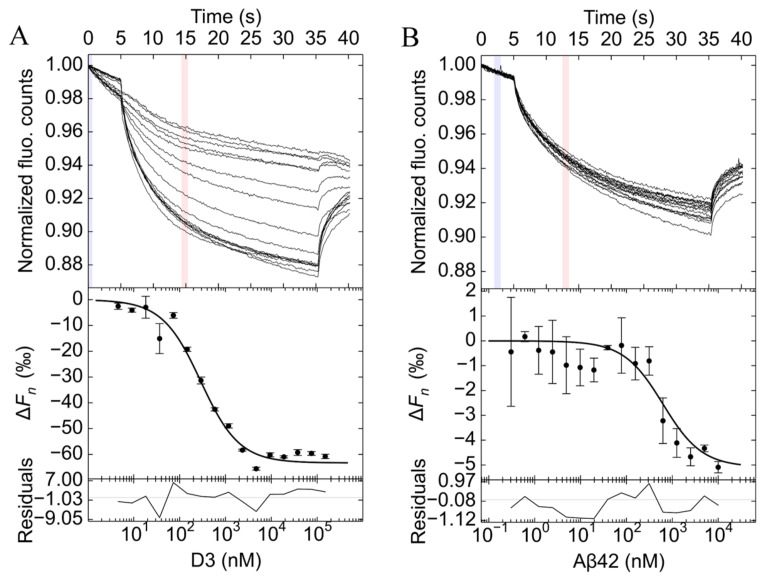
Microscale thermophoresis analyses to determine the dissociation constant of amyloid-β peptide (Aβ)42 and D3 interaction. Experiments with D3 and 40 nM fluorescein isothiocyanate (FITC)-Aβ42 (**A**), and the reciprocal approach with Aβ42 and 40 nM FITC-D3 (**B**), were conducted at 22 °C. Samples were prepared in 20 mM sodium phosphate and 50 mM NaCl (pH 7.4—slightly alkali). To minimize unspecific surface adsorption, 0.01% (*v*/*v*) Tween 20 (Tw20) in (**A)** and 0.0004% (*w*/*v*) polyethylenimine (PEI or polyaziridine) in (**B)** were used. Representative time traces from one measurement are shown. $∆F_{n}$ was calculated according to the reference zone (light blue) and the analysis zone (light red). Data was analyzed using the 1:1 binding model implemented in the PALMIST software with a 68.3% confidence interval. Residuals of the fitting are included at the bottom of each graph. Samples were prepared in triplicate.

The dissociation constant of Aβ42 and D3 interaction was evaluated using the solution-based MST. This method has been developed on the basis of the Soret effect (or thermophoresis), which describes the directed movement of particles in response to a temperature gradient in a fluid [17,18]. Particles with different sizes, structures or charges may exhibit different thermophoretic behaviors [19]. In this context we examined the thermophoresis of unbound and bound molecules through titrating D3 to fluorescein isothiocyanate (FITC)-Aβ42, or vice versa. The fluorophore alone that had been incubated with different concentrations of D3 did not show any thermophoretic response in any control experiments, implicating no direct interaction between D3 and fluorescein (Figure S1). A 1:1 binding model was applied to fit all of the data, resulting in reasonable fits, as judged from root-mean-square deviation (RMSD) values and residuals. As evident from Figure 1A, D3 binds to FITC-Aβ42 with a *K*_D_ of 270 [240, 310] nM (68.3% confidence interval). The positive thermophoretic response of FITC-Aβ42 and D3 samples suggests that the bound state had higher depletion compared with the unbound state. To study how ionic strength may impact the binding, we measured the *K*_D_ in a buffer with reduced ionic strength. As indicated in Figure S2, the binding between FITC-Aβ42 and D3 was enhanced about threefold in a low ionic strength buffer, with a *K*_D_ of 88 [82, 99] nM. 

Another set of experiments, using 40 nM FITC-D3 and various concentrations of unlabeled Aβ42, was performed to verify the dissociation constant (Figure 1B). The determined *K*_D_ for FITC-D3 and Aβ42 was 600 [400, 870] nM. This value is about twice of that one derived from the experiments with FITC-Aβ42 and D3 under the same conditions. However, considering the much smaller thermophoretic response in FITC-D3 samples than those in FITC-Aβ42 samples, the deviation between the two *K*_D_ values is generally within the range of precision. Due to the low concentrations of analytes in MST, the monomer binding should be the dominant reaction in solution. Therefore, MST data demonstrated that there is a strong interaction between flexible D3 and aggregation-prone Aβ42 monomers at nanomolar affinities, and that electrostatic effects play a role in the interaction.

### 2.2. AUC Analysis of Aβ42 and D3 Mixtures

**Figure 2:**
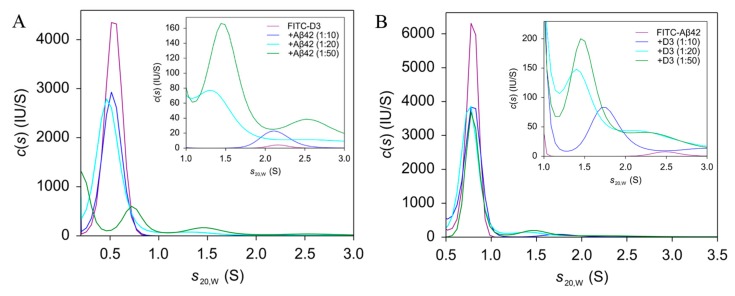
Sedimentation velocity analyses of 0.2 μM FITC-D3 incubated with different concentrations of Aβ42 (**A**), and 0.33 μM FITC-Aβ42 incubated with different concentrations of D3 (**B**), using fluorescence-based analytical ultracentrifugation (AUC-FDS). FITC-D3 samples were prepared in 20 mM sodium phosphate, 50 mM NaCl (pH 7.4—slightly alkali), 0.0004% (*w*/*v*) polyethylenimine (PEI) (also known as polyaziridine). FITC-Aβ42 samples were prepared in 55 mM Tris, 50 mM NaCl (pH 7.4), 0.01% Tw20. Note that PEI and Tw20 were used to overcome the unspecific surface adsorption. Samples were centrifuged at 60,000 rpm (289,000× *g*) for 15 h, and were analyzed with the *c*(*s*) model to determine sedimentation coefficient distributions. Inserts show the enlargement of the size distribution between 1 and 3 S. All *s*-values were standardized to *s*_20,w_.

To investigate the complexation between Aβ42 and D3, we performed sedimentation velocity measurements in an analytical ultracentrifuge using a fluorescence detection system. Aside from the high sensitivity, the advantage of the fluorescence detection system is the selectivity of detection, since unlabeled analytes are invisible [20,21,22]. The high sensitivity enables the measurement at analyte concentrations close to the determined dissociation constants in the nanomolar range. Among the two possible setups, experiments with FITC-D3 and unlabeled Aβ42 should possess a higher sensitivity for detection of a 1:1 complex than the reversed experiments, because the size difference between D3 and D3-Aβ42 is larger than that between Aβ42 and Aβ42-D3. The sedimentation coefficient (*s*_20,w_) for FITC-D3 was determined as 0.54 ± 0.02 S (Figure 2A). D3 itself does not form any self-assemblies or aggregates (see magenta curve in Figure 2A). The sedimentation velocity measurement confirmed that D3 stays monomeric in solution, since no other sedimenting species could be detected. 

At concentrations close to the dissociation constant, the addition of unlabeled Aβ42 to FITC-D3 should result in the appearance of a new sedimenting species, indicating either the sedimentation of hetero-complexes, or the sedimentation of a reaction boundary as found for rapid reactions [23,24]. Ideally, the total signal would stay constant irrespective of the added amount of unlabeled binding partner. Nevertheless, in the presence of excessive amounts of Aβ42, a significant fluorescence signal loss for FITC-D3 samples was noticed already during the FDS calibration process at 3000 rpm (726× *g*) (Figure S3). Corresponding to this, we observed from the *c*(*s*) analysis that the area under curve for the monomeric species around 0.5 S also showed an Aβ42-concentration-dependent reduction. We hypothesize that the observed signal loss is a consequence of the sedimentation of large aggregate species, which resulted from the further growth of small Aβ42-D3 complexes during the thermal equilibration process (~2 h at 3000 rpm during optics calibration). This could be supported by turbidity assays, showing that the addition of D3 to Aβ42 samples promotes the rapid formation of co-precipitates in a concentration-dependent manner (Figure S4). It can be seen from *c*(*s*) distributions for FITC-D3 and Aβ42 mixtures (Figure 2A) that the *s*_20,w_ of the main peak shifted from 0.5 S in FITC-D3 alone to 0.71 ± 0.04 S in samples with a 50-fold excess of Aβ42. This sedimentation coefficient cannot be interpreted as FITC-D3 monomer, since the monomer can only reach a theoretical maximum of 0.7 S under the assumptions of a perfect sphere and no hydration, according to its molecular mass and partial specific volume. Because D3 does not oligomerize by itself, it was concluded that D3 forms a 1:1 complex with the Aβ42 monomer. Additionally, we observed a small fraction of FITC-D3 sedimenting at *s*-values between 1 and 3 S (shown in the insert of Figure 2A), indicating low amounts of larger complexes. The averaged *s*_20,w_ for the new species, by integrating the peak between 1 and 2 S, is 1.45 ± 0.08 S, according to *c*(*s*) analyses from five independent experiments, suggesting the presence of complexes with higher stoichiometries. Although the detected amount of the newly formed species was rather low, we were able to observe an increase in the weight average *s*_20,w_ for FITC-D3 samples along with Aβ42 addition (Figure S5A).

The AUC analyses on FITC-Aβ42 and D3 exhibited similar *c*(*s*) distribution patterns to those from FITC-D3 and Aβ42 (Figure 2B). The FITC-Aβ42 monomer has an *s*_20,w_ of 0.77 ± 0.02 S and comprises more than 95% of the total signal of the sample solution. The monomeric nature of the sample persists throughout the measurement, permitting the study of any Aβ42 monomer-D3 interactions. As mentioned, the FITC-Aβ42 monomer has an *s*_20,w_ of 0.77 ± 0.02 S, which impedes the observation of the 1:1 complex of D3 and FITC-Aβ42, which can be expected to be very close to 0.71 S. Incubating D3 with FITC-Aβ42 led to the formation of new species at 1.54 ± 0.11 S in a D3 concentration-dependent manner. Besides, the weight average *s*_20,w_ of FITC-Aβ42 and D3 mixtures increased gradually with an increasing D3 concentration (Figure S5B). Since the *c*(*s*) distribution of Aβ42 alone did not show any peak between 1 and 2 S [25], the new species are most likely hetero-complexes of FITC-Aβ42 and D3 at stoichiometries higher than 1:1, rather than Aβ42 homo-oligomers. Both sets of AUC experiments, using FITC-D3 and Aβ42, as well as FITC-Aβ42 and D3, clearly validate the interaction between Aβ42 monomers and D3. The results revealed for the first time the complex formation between highly flexible and aggregation-prone Aβ42 monomers and unstructured D3 peptides at variable stoichiometries.

### 2.3. Complex Formation Studied by Molecular Dynamics Simulation

**Figure 3:**
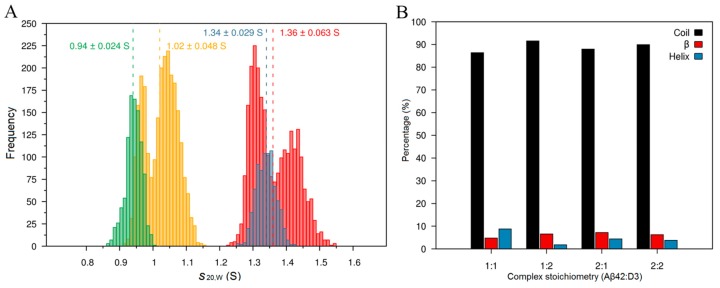
Sedimentation coefficient (*s*_20,w_) distribution of D3 and Aβ42 monomer complexes (**A**) and calculation of the secondary structure of Aβ42 monomers in these complexes (**B**) based on MD simulations. The average *s*_20,w_ with standard deviations for the 1:1 (green), 2:1 (yellow), 1:2 (blue) and 2:2 (red) D3:Aβ42 complexes are given for each complex above the corresponding histograms. Random coil, turns and bends are denoted as coil structures, while β-sheets and β-bridges are known as β-structures, and finally, α-, 3_10_- and π-helices, as helix structures.

We performed MD simulations to gain further insights into the complexation between Aβ42 and D3. The different complexes formed between 20 Aβ42 and five D3 molecules during the simulation were used to derive theoretical sedimentation coefficients for different stoichiometries, focusing on four stoichiometries, namely 1:1, 1:2, 2:1 and 2:2 (D3:Aβ42), which had *s*-values close to the experimentally-observed values (Figure 3 and Figure S6). The 1:1 and 1:2 complexes were calculated to have *s*_20,w_ at around 1 S, while the 2:1 and 2:2 complexes were calculated to have values at 1.3 to 1.4 S. We also calculated *s*-values for larger complexes observed in the initial implicit solvent simulations, and calculated an *s*_20,w_ of 1.6 to 1.7 S for 1:3, ~1.7 S for 2:3 and ~1.9 S for 1:4 stoichiometries (D3:Aβ42). The calculated *s*-value for the 1:1 complex in MD simulations corroborated AUC results that D3 forms 1:1 complexes with Aβ42, while the new species observed at ~1.45 S in AUC measurements with FITC-D3, and the high excess of Aβ42 does result most likely from complexes with higher stoichiometries (for instance, 1:2 D3:Aβ42). In turn, the new species observed at ~1.54 S in AUC measurements with FITC-Aβ42 and the high excess of D3 is most likely to be the 2:1 or 3:1 (D3:Aβ42) complex.

The lack of a fixed stoichiometry in the complexation between D3 and Aβ42 led us to think whether the interaction induces significant structural changes in disordered Aβ42 monomers or not. We then analyzed the secondary structure of Aβ42 in all four complexes obtained in the simulation. Aβ42 monomers remained predominantly unstructured, with less than 10% β-structures in all complexes (Figure 3B); this indicates that the interaction between D3 and Aβ42 monomers is rather flexible and dynamic.

Results from MD simulations are in line with fluorescence AUC measurements, and implicate that the interaction between D3 and Aβ42 monomers is analogous to the dynamic interaction between two IDPs.

### 2.4. D3 Retards the Fibrillation of Aβ42 at Substoichiometric Concentrations

**Figure 4:**
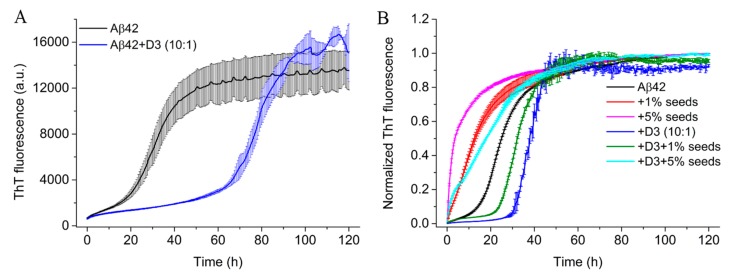
ThT assays showing fibrillation kinetics of 20 μM Aβ42 and 20 μM Aβ42, with 2 μM D3 in (**A**), and of 10 µM seeded Aβ42 (1% or 5% seeds), incubated without or with 0.1-fold D3 in (**B**). Color usage is explained in the figure. Samples were incubated in 20 mM sodium phosphate, 50 mM NaCl (pH 7.4—slightly alkali) at 20 °C. ThT data is averaged based on samples prepared in triplicate.

The aggregation kinetics of Aβ42 as observed in the ThT assay generally displays a sigmoidal pattern characterized by three phases: The lag phase, the rapid growth phase and the plateau phase [26,27,28]. In the primary nucleation, which is the limiting event during lag phase, Aβ monomers associate and form aggregates (primary nuclei) without the involvement of already formed assemblies [28]. During rapid growth or elongation Aβ monomers are added to the ends of already formed aggregates (such as nuclei), leading to fibrillar structures. Additionally, once formed fibrillar structures provide surfaces at which monomers can be catalyzed to form nuclei. This process is called secondary nucleation [29]. Finally, a plateau is reached due to the consumption of free monomers [28,30]. At high concentrations of D3 the fibril formation as monitored by ThT can be largely suppressed (data not shown). However, here we used only 0.1-fold D3 concentration to render the measurements comparable to the CD measurements. As shown in Figure 4A and Table 1, Aβ42 alone displayed a sigmoidal fibrillation kinetics and an earlier onset of rapid growth than Aβ42 incubated with D3. The $t_{1/2}$ and $t_{lag}$ of Aβ42 alone were determined to be 30.0 ± 0.7 h and 17.3 ± 1.6 h, respectively, according to the fitting (Figure S7). Addition of 0.1 equimolar D3 slowed down the fibril formation of Aβ42 significantly, as $t_{1/2}$ and $t_{lag}$ were prolonged to 79.3 ± 2.3 h and 67.6 ± 3.8 h, respectively. We also noticed that the growth phase seemed to be unaffected, as both samples had similar slopes. All samples reached comparable plateau fluorescence signals after 120 h of incubation irrespective of the addition of D3. Thus, D3 effectively elongates the lag phase of Aβ42 aggregation, very similar to what was described for bexarotene [31]. To figure out whether or not the interaction between D3 and Aβ42 was affected by the buffer system, we performed ThT measurements in Tris-HCl buffer with the same ionic strength as the phosphate buffer. A comparable ThT kinetics was observed in both buffers (Figure S8 and Table S2), suggesting that the retardation effect of D3 on Aβ42 aggregation is not very dependent upon the buffer system. The difference in the ThT kinetics of Aβ42 with or without D3 hints that samples might have different aggregate compositions. We therefore performed AUC experiments on equivalent samples incubated for 24 h. At 24 h, Aβ42 alone should have ThT positive species, while Aβ42 plus D3 should still be in the lag phase. Samples of Aβ42 alone contained a fraction of large aggregates, which were sedimented to the cell bottom during the acceleration process of AUC (Figure S9), these presumably being products of the elongation phase. In contrast, Aβ42 samples with D3 had no such large aggregates, but a significantly higher amount of monomers (~0.7 S) than Aβ42 alone (Figure S10). The dramatic difference in the size distribution of Aβ42 with or without D3 suggests that D3 is able to interfere with the very early stage of Aβ aggregation, which is the nucleation process, by retaining Aβ monomers and delaying the growth and amplification of Aβ nuclei.

To further investigate how D3 may interfere with the aggregation process of Aβ42, we performed seeding experiments. In these experiments, the introduction of fibril fragments generated by sonication into Aβ monomer solutions offers surfaces and/or fibril ends for the attachment of monomeric species, leading to an immediate growth of fibrillar structures [32,33]. The presence of Aβ42 seeds significantly accelerated the aggregation of Aβ42 by canceling the lag phase, as evident from ThT kinetics (Figure 4B). In particular, the addition of 5% seeds in Aβ42 samples immediately initiated the rapid ThT fluorescence increase of Aβ42 aggregation. The inhibitory effect of D3 on the fibrillation was also visible in Aβ42 samples incubated with seeds. Aβ42 samples with both D3 and 1% seeds had a longer lag phase than Aβ42 without D3 (Figure 4B). Although D3 at 0.1-fold was not able to restore the sigmoidal aggregation kinetic of Aβ42 in the presence of 5% seeds, we could still observe a retardation of the elongation process in samples with D3 treatment, by comparing the slopes of the ThT kinetics with those of Aβ42 samples incubated solely with 5% seeds (Figure 4B), suggesting that substoichiometric D3 could interfere with the elongation of Aβ42 fibrils. ThT kinetics indicated that D3 may also interact with aggregated Aβ42 species (such as oligomers and fibrillar structures).

### 2.5. D3 Slows Down the Secondary Structure Conversion of Aβ42 at Substoichiometric Concentrations

**Figure 5:**
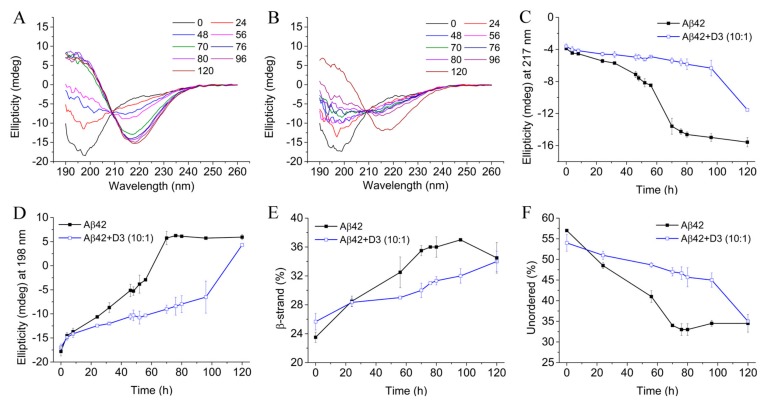
Circular dichroism (CD) measurements and spectrum deconvolution analyses. 40 μM Aβ42 was incubated with (**A**) or without (**B**) 4 μM D3 in 20 mM sodium phosphate, 50 mM NaF (pH 7.4—slightly alkali) at 20 °C. CD spectra were recorded at indicated time points between 0 and 120 h of incubation. Transition kinetics are shown by plotting ellipticities at 217 nm (**C**) and 198 nm (**D**) against the incubation time. Changes in fractions of β-strand (**E**) and unordered structures (**F**) over the incubation time were obtained using the CDSSTR algorithm and reference dataset 7 in Dichroweb. All samples were prepared in triplicate.

The fibrillation of Aβ42 is accompanied by a structural transformation of Aβ42 monomer from random coil to β-sheet structures. D3 itself does not have stable secondary structure elements, and is disordered in solution (Figure S11). The addition of low amounts of D3 did not contribute detectably to the overall spectra of Aβ42 samples, so that the structural conversion of Aβ42 was observable without requiring correction for the D3 signal. Equally important, the low D3 concentration avoided the formation of co-precipitates with Aβ42, which might impede measurements. As shown in Figure 5A, Aβ42 alone adopted mainly random coil structure at the beginning, and converted to β-sheet conformation during the incubation at 20 °C. The transition kinetics of Aβ42 alone based on incubation time-dependent ellipticities at 217 nm and 198 nm exhibited sigmoidal patterns for both wavelengths (Figure 5C,D). However, Aβ42 treated with D3 alone had markedly different transition kinetics, as can been seen from Figure 5B–D. The overall transition kinetics of 40 μM Aβ42 was significantly delayed in the presence of 4 μM D3. The ellipticity at 217 nm for the Aβ42 sample with D3 was about 72% of that for Aβ42 alone at 120 h, pointing to decreased β-sheet structures in samples with D3. Dichroweb deconvolution of CD spectra to evaluate secondary structure components of samples incubated with or without D3 demonstrated similar trends (Figure 5E,F). There was a gradual loss of unordered conformations accompanied by accumulations of β-strand structures in all samples. Nevertheless, the free Aβ42 sample had faster conversion rates than Aβ42 samples containing 0.1-fold D3. The fractions of β-strand structures in Aβ42 samples without D3 addition were also higher than those in samples incubated with D3 at most of the time points. Even, if 4 μM D3 was added into 40 μM Aβ42 after a preincubation period of 33 h (Figure S12), it still visibly delayed the structural transformation of Aβ42. The results from CD measurements are in agreement with those obtained from ThT assays in demonstrating that substoichiometric D3 decelerates the fibril formation process of Aβ42 via retarding the secondary structure conversion. The data also offers experimental evidence to corroborate MD simulations that D3 favors a less structured state of Aβ42.

### 2.6. Morphologies of Aβ42 Samples in the Presence of Substoichiometric D3

**Figure 6:**
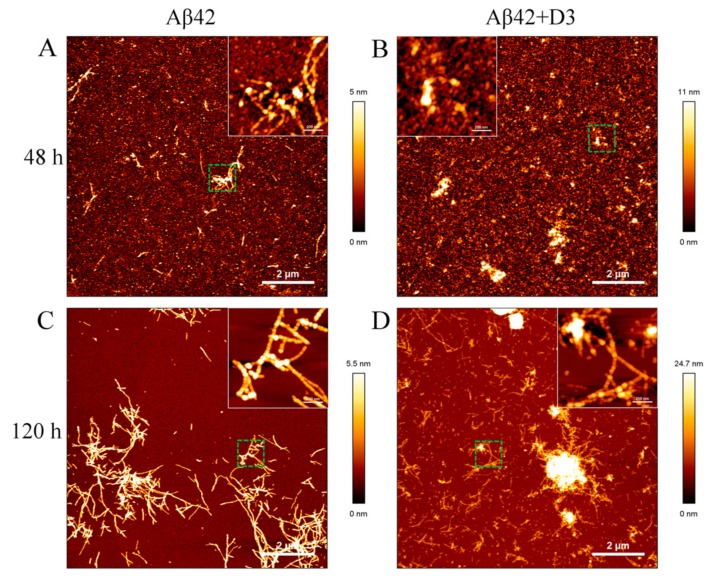
Atomic force microscopy (AFM) imaging to study the morphologies of 40 μM Aβ42 in the absence (**A** and **C**) and presence (**B** and **D**) of 4 μM D3 in 20 mM sodium phosphate, 50 mM NaCl (pH 7.4—slightly alkali) at 20 °C after 48 h and 120 h of incubation. The imaging was carried out at room temperature in air, using AC mode. Inserts show amplifications of areas marked with green squares. The scale bars are 2 μm for the overviews and 200 nm for the inserts.

AFM imaging was conducted to detect the morphologies of Aβ42 samples incubated with or without D3 for 48 h and 120 h. According to ThT assays and CD measurements, Aβ42 alone should have a considerable amount of fibrillary structures after incubating for 48 h. This was confirmed by AFM imaging, which showed rodlike fibrillar structures in Aβ42 alone after 48 h incubation, as displayed in Figure 6A. The typical height for single fibrils was around 5 nm. Some protofibrils and oligomers (~2 nm in height) were also visible in Aβ42 alone after 48 h of incubation. However, Aβ42 incubated with 0.1 equimolar D3 had no fibrils, but some amorphous precipitates or aggregates with heights varying from 10 nm to 20 nm (Figure 6B). Small species with similar dimensions to protofibrils and oligomers in Aβ42 alone were also present. After 120 h of incubation, most of the materials transformed to amyloid fibrils in free Aβ42 samples, as shown in Figure 6C. These fibrils did not differ much from Aβ42 fibrils found at 48 h with respect to the height, but became more elongated, which agrees well with the findings of Arimon et al. [35]. Substoichiometric D3 did not stop the fibril formation, as we could see rod-like fibrils as well in Aβ42 incubated with D3 for 120 h (Figure 6D). Surprisingly, amorphous aggregates also grew to larger size than those seen at 48 h. AFM imaging provided additional evidence that D3 affects the aggregation process of Aβ42 at substoichiometric levels. If studied for cytotoxicity, such amorphous co-aggregates or co-precipitates have always been non-toxic in cell culture, as reported previously [13,36].

## 3. Discussion

The present study reports how two IDPs, the all-D-enantiomeric peptide D3 and Aβ42 monomers interact, and how the interaction may influence the fibrillation of Aβ42. We chose solution-based methods in combination with fluorescence-based detection in order to minimize the required concentrations of analytes as well as surface-related effects. The binding affinity between D3 and Aβ42 was evaluated using MST in both possible setups. Smooth capillary scans, as well as the reproducibility of the determined dissociation constant in successive rounds of measurements, indicated the absence of irreversible aggregation. A simple 1:1 binding model was sufficient for fitting the data from both setups. Other more complex models did not significantly improve the fit. The *K*_D_ for D3 and Aβ42 at about 400 nM is about 10-fold smaller than that reported in our previous SPR studies (~4 μM) [13,37]. Possibly, the fixation of n-terminally biotinylated Aβ42 monomers onto sensor chips via biotin–streptavidin coupling rendered the interaction between D3 and Aβ42 monomers less efficient than that found for both components free in solution [13]. Lowering the ionic strength in MST measurements increased the *K*_D_, pointing to the involvement of electrostatic interactions between the positively-charged D3 and the negatively-charged Aβ42. Ionic strength may affect protein-protein interactions in many ways, e.g., a reduction in the salt concentration may cause a decrease in the screening effect of salt-ions near the protein surface [38], thus enhancing the electrostatic attraction between oppositely-charged proteins, and increasing the binding affinity. This effect is confirmed by Borgia et al. who found that reducing the ionic strength strongly promotes the complex formation between two IDPs carrying opposite charges [16]. Our findings agree well with a study on a decapeptide containing three L-Arg residues, in that reducing the ionic strength of the buffer strengthens the interaction between ligands and Aβ40 monomers [39]. This feature of D3 and Aβ42 interaction also coincides well with the interaction between two typical IDPs, in which the electrostatic effect usually has an important contribution [40,41].

AUC analyses from both FITC-Aβ42 and FITC-D3 samples demonstrated that binding between D3 and Aβ42 leads to the formation of new species sedimenting faster than the corresponding monomer, which represent most of the probably small hetero-complexes. Albeit a 1:1 hetero-complex could only be clearly detected in FITC-D3 samples treated with Aβ, an equivalent new species is most probably also present in the case of FITC-Aβ42 with D3, but its expected *s*-value cannot be distinguished from the *s*-value of free FITC-Aβ42. In the case of a small change in mass upon complex formation, 5 kDa (FITC-Aβ42) versus 6.6 kDa (FITC-Aβ42+D3), unfavorable conditions like an extended shape upon complex formation, or high dissociation rates, can cause the complex to sediment at reduced speed, resulting in a sedimentation coefficient of the 1:1 complex indistinguishable from that of the free monomer. Nevertheless, the increase in weight average *s*-values in both groups of samples confirmed the complexation between D3 and Aβ42 monomers. It is evident from AUC analyses on individual proteins that monomers are the dominant species in Aβ42 or D3 samples at the nanomolar concentrations used in the study. Therefore, the monomer interaction is the major event under the hereby applied experimental conditions. Similar findings were also reported by Cox et al., showing that small heat shock proteins can transiently interact with α-synuclein monomers and prevent its aggregation [42]. Through MD simulations, we have identified four possible complexes of D3 and Aβ42 within the *s*-value range of AUC experiments. The higher stoichiometry complexes may therefore evolve from the further interaction between 1:1 complexes and additional D3 or Aβ42 molecules in solution. Indeed, the reduction in the amount of monomeric species in both AUC setups points to the fact that D3 or Aβ42 monomers have been consumed in the presence of their binding partners.

The initial step of Aβ42 fibril growth is the formation of nuclei which serve as seeds for the further growth of amyloid fibrils. By complementing ThT kinetics with AUC experiments, we were able to monitor the complete composition of Aβ samples including ThT negative species, such as monomers and small oligomers. The much higher content of Aβ42 monomers and small oligomers in samples with substoichiometric D3 than in samples without D3 explains the much slower aggregation and structural transformation kinetics of D3-containing samples. It seems that interacting with D3 impedes the formation of any Aβ oligomers required for seeding the fibrillation. The outcome of D3 affecting the aggregation kinetics of Aβ42 is similar to results obtained by Assarsson et al., showing that the small hydrophilic proteins, calbindin D_9k_, and single-chain monellin, retard the fibril formation process of Aβ40 in a net charge-dependent manner. They found that proteins with positive or low negative net charges are particularly effective in slowing down the fibrillation [43]. Interestingly, we also observed in seeding experiments that substoichiometric amounts of D3 retard the fibril formation of Aβ42 efficiently, even in the presence of a relatively high amount of seeds. AUC and MST measurements demonstrated that D3 directly interacts with Aβ42 monomers. Sequestration of monomers by 0.1-fold D3 would decrease the available Aβ42 concentration by 10%. Although aggregation is concentration dependent, a difference in ThT kinetics for 18 µM versus 20 µM Aβ42 was not detectable (data not shown). Therefore the formation of the stable 1:1 complex alone is insufficient to explain the diverse effects of D3 on the fibrillation of Aβ42 at substoichiometric concentrations. Given the highly flexible nature of small D3-Aβ42 monomer complexes revealed by MD simulations, which is very similar to what has been reported for the transient interaction for the chaperonin GroEL and Aβ42 monomer [44], the simplest explanation may be that the D3 forms transient complexes with Aβ monomers, rendering them fibrillation-incompetent and interfering with the nucleation process. This would allow D3-mediated stabilization of Aβ monomers and inhibition of amyloid growth even at substoichiometric concentrations.

Intriguingly, the seeding ThT kinetics showed that both the lag phase and the growth phase of fibril formation can be affected by D3. A possible explanation could be that D3 binding the seeds renders those seedings incompetent. Although one might argue that retarding the elongation of Aβ42, particularly by interacting with fibril ends, might lead to an increase in the generation of toxic oligomers catalyzed by secondary nucleation [29], we have shown in animal studies that D-peptides actually reduced the amount of toxic oligomers in the central nervous system and conferred protective effects [37]. It can be seen from the ThT kinetics of Aβ42 incubated with both D3 and seeds that the aggregation process of Aβ42 displayed a biphasic pattern in these samples, i.e., samples showed an initial increase in ThT fluorescence to a first plateau, followed by a second rapid increase in ThT fluorescence. 

The biphasic aggregation kinetics has been observed for Aβ at relatively high protein concentrations [45,46], and is likely due to the formation of metastable globular oligomers and curvilinear fibrils in the first phase, which act as off-pathway inhibitors of Aβ fibrillation [47]. A possible explanation might be that D3 can not only bind to Aβ42 monomers and preserve their disordered conformations, but also transiently interacts with Aβ42 subunits in Aβ42 assemblies, such as oligomers and fibrillar structures. The presence of D3 may redirect Aβ42 seeds to off-pathway species, thus canceling their seeding capabilities. These mechanisms play a role in a D3-mediated deceleration of Aβ fibrillation, and could complement the interaction of D3 with Aβ monomers.

ThT assays are corroborated by CD measurements, showing that D3 is effective in retarding the secondary structure conversion of Aβ42 to β-sheet structures. The transformation of unordered Aβ42 monomers to β-sheet rich structures is critical to the fibrillation, since they function as building blocks for amyloid fibrils [48]. The result is consistent with a previous MD analysis showing that D3 disrupts the formation of β-sheet structures in Aβ42 by binding adjacently to the n-terminal half of Aβ42, where the central hydrophobic core is located [49]. In fact, the content of β-sheet structures in Aβ42 and D3 complexes (~5%) is much lower than that observed for the Aβ42 dimer (~15%) in previous simulations in the same force field [50]. The rather unordered structure of Aβ42 monomers in complex with D3 makes them incapable of participating in Aβ nuclei growth and amplification processes encompassing the accumulation of a certain amount of ordered structures [51,52,53]. The experimental data, together with MD simulations, also reveal that when bound to D3, Aβ42 maintains unordered structures similar to those in free solution. To our knowledge, this is the first experimental evidence that D3 directly influences the structural conversion of Aβ42 at substoichiometric concentrations, most probably by transiently forming complexes with Aβ monomers and small assemblies. The morphologies obtained using AFM reflects exactly what we have concluded from ThT and CD measurements, that the growth of fibrillary structures is greatly delayed in the presence of D3. The amorphous Aβ42 aggregates formed in the presence of substoichiometric D3 implicate that the complexation between D3 and Aβ42 triggers further aggregation to form large co-precipitates, at least under the unphysiologically high Aβ concentrations. Previous studies revealed that these amorphous co-precipitates are non-toxic species in cell culture assays [36]. We have, however, never observed the formation of large amorphous precipitates under physiological conditions, where the concentration of Aβ is at the low nanomolar level. This result is consistent with previous findings that D3 reduces Aβ oligomer-mediated toxicity, and therefore has beneficial effects on cognition in vivo [13,36].

## 4. Materials and Methods

### 4.1. Chemicals and Reagents

Fluorescein isothiocyanate (FITC)-labeled amyloid-β peptide (Aβ)42 protein (product No. M-2585.1000) was purchased from Bachem (Weil am Rhein, Germany). The conjugation was made via one additional β-Alanine (β-Ala) at the N-terminus of the Aβ42 sequence (FITC-β-Ala-Aβ42, hereinafter referred to as FITC-Aβ42). According to the manufacturer, the purity of the product is 88.2%. Unlabeled Aβ42 protein (product No. H-1368.1000) was also obtained from Bachem with a purity of 95.2%, as determined by high-performance liquid chromatography (HPLC). Aβ42 products (1 mg) were first dissolved in 100% 1, 1, 1, 3, 3, 3-hexafluoro-2-propanol (HFIP) overnight to monomerize the materials. The solutions were then divided into aliquots, and were lyophilized to evaporate HFIP. Proteins were stored at −80 °C until their use. C-terminally amidated D3 (H-rprtrlhthrnr-NH2) was available from peptides & elephants (Hennigsdorf, Germany) as lyophilized powder with >95% purity. The fluorescently-labeled D3 from the same manufacturer was prepared via conjugating 5(6)-carboxyfluorescein with an additional l-lysine (Lys) residue at the C-terminus of D3. The purity for the product was determined to be 98% in HPLC tests. The stock solutions of D3 were prepared with H_2_O, and were diluted to working concentrations with either 20 mM sodium phosphate, 50 mM NaCl (pH 7.4—slightly alkali) or 55 mM Tris-HCl, 50 mM NaCl (pH 7.4—slightly alkali), depending upon the experiment. 

Tween-20 (Tw20, 0.01%, *v*/*v*) and polyethylenimine solution (PEI (also known as polyaziridine), branched, average Mw ~1300, 0.0004%, *w*/*v*) were used to attenuate surface adsorption of FITC-Aβ42 and FITC-D3 in fluorescence-based measurements in the present study, respectively.

A 1 mM stock solution of thioflavin T (ThT) was prepared in H_2_O and sterile-filtered before use to remove any particles that may influence the aggregation of Aβ42.

### 4.2. Microscale Thermophoresis

The dissociation constant of Aβ42 and D3 interaction was characterized by microscale thermophoresis. In detail, FITC-Aβ42 was dissolved in 20 mM sodium phosphate, 50 mM NaCl (pH 7.4—slightly alkali), 0.01% (*v*/*v*) Tw20 to obtain 80 nM stock solutions. 300 μM D3 was prepared in the same buffer. FITC-Aβ42 was then titrated with D3 solutions in 1:1 serial dilution steps to prepare 16 samples, in which the concentration of FITC-Aβ42 was kept constant at 40 nM, and the starting concentration of D3 was 150 μM. Samples were loaded into standard capillaries, and the thermophoresis was detected using a Monolith NT.115 (NanoTemper Technologies GmbH, Munich, Germany). The experiment was performed with 40% LED power and 60% microscale thermophoresis (MST) power. In order to understand how ionic strength influences the interaction between Aβ42 and D3, MST the measurement was repeated in 5 mM sodium phosphate, 50 mM NaCl (pH 7.4—slightly alkali), 0.01% (*v*/*v*) Tw20 under the same conditions. For experiments with FITC-D3 and Aβ42, 80 nM FITC-D3 was mixed with different concentrations of Aβ42 in 20 mM sodium phosphate, 50 mM NaCl (pH 7.4—slightly alkali), 0.0004% (*w*/*v*) PEI to prepare a concentration series with Aβ42 concentration starting at 10 μM. The final concentration of FITC-D3 in each sample was set to 40 nM. The LED power and MST power were adjusted to 30% and 60%, respectively. The on and off time for the IR laser was set to 30 s and 5 s. Since the working concentration of labeled molecules was rather low in this measurement, any signal loss due to surface adsorption might bias the data acquisition. Therefore trace amounts of Tw20 and PEI were used as additives in solution to minimize the unspecific surface adsorption of FITC-Aβ42 and FITC-D3 in capillaries, respectively. All measurements were conducted at 22 °C, and samples were prepared in triplicate.

In order to exclude that a direct interaction between D3 and the fluorophore contributes to the observed signals, control experiments with fluorescein alone titrated with D3 at the same concentrations as applied for FITC-Aβ42 were performed.

The data was evaluated with PALMIST software (version 1.2.3) to obtain the dissociation constants [54]. Changes in the normalized fluorescence ($∆F_{n}$) with concentrations of the titrant were quantified and fitted with the 1:1 binding model available in the software using a 68.3% confidence interval. The graphic outputs were created using GUSSI (version 1.2.1) [55].

### 4.3. Analytical Ultracentrifugation

Sedimentation velocity analysis was conducted to evaluate size distributions of Aβ42 and D3 mixtures. All measurements were performed using an XL-A analytical ultracentrifuge (Beckman coulter, Brea, CA, USA). For the determination of a complex formation between D3 and Aβ42, a fluorescence detection system (Aviv Biomedical Inc., Lakewood, NJ, USA) was used. FITC-D3 at 0.2 μM was incubated with different concentrations of Aβ42 to prepare mixtures at molar ratios of 1:10, 1:20 and 1:50 (D3:Aβ42). All samples were prepared in 20 mM sodium phosphate, 50 mM NaCl (pH 7.4) containing 0.0004% PEI (*w*/*v*). Samples were then loaded into 3-mm double-sector titanium cells (Nanolytics, Potsdam, Germany), with each sector containing a 100 μL sample. The detection system uses an excitation laser at 488 nm and an emission cut-off filter at 505 nm to collect fluorescence signals. The amplification factor was adjusted to the same value for all samples for comparability. To complement, 0.33 μM FITC-Aβ42 was incubated with or without various concentrations of D3 in 55 mM Tris-HCl, 50 mM NaCl (pH 7.4), 0.01% Tw20, and samples were analyzed under the identical condition to the AUC measurements on FITC-D3 samples. Data was acquired at 20 nm radial resolution. After thorough thermal equilibration the centrifugation was carried out at 60,000 rpm (289,000× *g*) at 20 °C for 15 h. Carrier proteins, such as albumin and kappa casein, are usually recommended to minimize unspecific surface adsorption for fluorescence-based AUC [56]. 

They are not suitable here, since both proteins are found to interact with Aβ42 [57,58,59]. Therefore, either PEI or Tw20 was included to suppress the surface adsorption of labeled molecules during the sedimentation.

An additional absorbance-based AUC was performed to check the size distribution of 20 μM Aβ42 treated with or without 2 μM D3 for 24 h. In brief, 380 μL samples were loaded into 12-mm double-sector aluminum cellsm and were thermally equilibrated prior to the final centrifugation. The centrifugation was performed at 45,000 rpm, 20 °C for 15.5 h. Sedimentation profiles were recorded at 210 nm, with a radial resolution of 20 μm.

All sedimentation profiles were subjected to the software package SEDFIT (version 15.01b) for data evaluation. The data was analyzed with the continuous distribution *c*(*s*) Lamm equation model to obtain the sedimentation coefficient distributions [60]. Fitting parameters (Table S1) including the buffer density and viscosity were determined using Sednterp (version 20130813BETA). Partial specific volumes for labeled and unlabeled Aβ42 proteins and D3 were calculated according to Sednterp and Durchschlag et al. [61,62]. Final graphs were generated using GUSSI (version 1.2.1) [55] and sedimentation coefficients were standardized to *s*-values in pure water at 20 °C (*s*_20,w_).

### 4.4. Molecular Dynamics (MD) Simulation and Data Analysis

#### 4.4.1. Simulation Setup

To obtain complexes formed by Aβ42 and D3, we performed five independent molecular dynamics (MD) simulations of twenty Aβ42 proteins and five D3 molecules in implicit solvent. The peptides were randomly placed in a simulation box with edge lengths of 41 nm × 33 nm × 38 nm and simulated for 100 to 325 ns using the parallel processing MD software Gromacs 4.5.5 [63]. The five simulations were initiated with different initial velocity distributions, but all corresponding to a temperature of 310 K. The OPLS/AA force field [64,65] was used to describe all peptides, and a Generalized Born model with a hydrophobic solvent accessible surface area term (GBSA) [66] represented the aqueous environment. The dynamics was integrated with a leap-frog stochastic dynamics algorithm, and periodic boundary conditions were applied. Hydrogen atoms were treated as virtual interaction sites, permitting an integration time step of 4 fs while maintaining energy conservation [67]. The temperature was kept at 310 K using velocity rescaling with a stochastic term algorithm [68] and a time constant for coupling of 2 ps. The electrostatic interactions were treated with a cut-off method with a value of 1.2 nm, and the van der Waals interactions were also cut at 1.2 nm. Snapshots were saved every 20 ps during each of the five MD simulations, from which all Aβ42-D3 complexes with stoichiometries of 1:1, 1:2, 2:1, and 2:2 were extracted from simulations and clustered, using the method of Daura and coworkers, with a cut-off of 0.2 nm [69]. For each stoichiometry, the five most populated conformations were selected as starting structures for the subsequent MD simulations with explicit solvent.

The explicit solvent MD simulations were performed with Gromacs 2018 [70], using the OPLS/AA force field [64,65] and TIP3P water model [71]. Each of the 4 × 5 complexes (i.e., 5 conformations for each of the 4 stoichiometries considered) was placed in a dodecahedron box containing 3000–5000 water molecules, and the resulting model system neutralized by adding the needed amount of Na^+^ or Cl^-^ ions. Before starting the production MD simulations, the energy of the systems was minimized until a maximal force of 100 kJ mol^−1^ nm^−1^ was reached with the steepest descent method. For further equilibration, MD simulations with restraints with a force constant of 1000 kJ mol^−1^ nm^−2^ on the heavy atoms of the peptides were performed for 50 ps under isothermal-isobaric (NPT) conditions with a temperature of 310 K and a pressure of 1 bar, using a velocity rescaling thermostat to regulate the temperature, and a Berendsen barostat for pressure control [72]. Another 20 ns of equilibration without restraints followed, before the production MD runs of 500 ns were executed. These simulations were also run under NPT conditions, but using an isotropic Parrinello-Rahman barostat for pressure control [73]. In all of these MD simulations, the electrostatic interactions were calculated via the particle mesh-Ewald method [74,75] in connection with periodic boundary conditions. 

The cutoff values for the van der Waals and short-range Coulombic interactions were set at 1.0 nm. The LINCS algorithm was used to constrain all bond lengths [76] and the hydrogen atoms were treated as virtual interaction sites, allowing us to apply an integration time step of 4 fs while maintaining energy conservation [67]. Snapshots were saved every 20 ps in each of the twenty 500 ns MD simulations.

#### 4.4.2. Prediction of the Sedimentation Coefficient

For determining the sedimentation coefficients of the simulated complexes, we first combined all snapshots collected during the 5 × 500 ns per stoichiometry, and then clustered them using the Daura algorithm [69] with a cut-off of 0.2 nm. For all resulting clusters, the sedimentation coefficient was calculated for the representative cluster conformation using the program HydroPro10 [77]. The partial specific volume was estimated by dividing the total volume of the Aβ42:D3 complex in question by the molecular weight of that complex, employing the 3V Volume Calculator for the volume, and Visual Molecular Dynamics (VMD) for the molecular weight [78,79]. For each Aβ42:D3 stoichiometry the mean sedimentation coefficient, along with the standard deviation, was determined by averaging over all clusters obtained for the 1:1, 1:2, 2:1 and 2:2 stoichiometry, respectively.

#### 4.4.3. Secondary Structure

The influence of complexation with D3 on the secondary structure of Aβ42 was characterized using the DSSP (Define Secondary Structure of Proteins) algorithm with default settings [80]. This method defines the secondary structures on the basis of energy calculations of H-bridges in the protein backbone. The average secondary structure of Aβ42 per complex stoichiometry was determined, where β-sheet and β-bridge were treated as β-structures, α-helix and 3_10_-helix regarded as helix structures, and random coil, bends and turns collectively considered as coil structures.

### 4.5. ThT Assay

Aβ42 at 20 μM was incubated with or without 2 μM D3 in 20 mM sodium phosphate, 50 mM NaCl (pH 7.4—slightly alkali). In parallel, 20 μM Aβ42 containing 2 μM D3 was prepared in 55 mM Tris-HCl, 50 mM NaCl (pH 7.4) retaining the ionic strength of the phosphate buffer to determine whether the effect of D3 depends on buffers. ThT was added at a final concentration of 5 μM in all samples. The final volume of each sample was 200 μL. Samples were prepared on ice and were pipetted into a 96-well plate afterwards. The plate was sealed with a microplate sealing film. All measurements were conducted at 20 °C with a microplate reader (Infinite M200, Tecan, Männedorf, Switzerland). The fluorescence was recorded at an excitation wavelength (λ_ex_) of 445 nm and an emission wavelength (λ_em_) of 485 nm every 30 min for 120 h. D3 alone does not induce ThT fluorescence. All samples were prepared in triplicate.

The ThT data was then subjected to the online server AmyloFit [34] with a customized sigmoidal Equation (1) to determine the lag time $t_{lag}$, and the half completion time of the aggregation process $t_{1/2}$, according to previous studies [81,82].

(1) $$I_{t}=k_{0}t+A/\left(1+\exp\left(-k\left(t-t_{1/2}\right)\right)\right),$$

where $k_{0}$ represents the slope of the baseline, A is the amplitude, k denotes the apparent elongation rate constant. The lag time $t_{lag}$ can be derived from the intercept between the time axis and the tangent with slope k from the midpoint of the fitted sigmoidal curve, which is given by the following Equation (2):

(2) $$t_{lag}=t_{1/2}-2/k$$

### 4.6. Seeding Experiment

Aβ42 seeds were prepared from fibrils according to Ehrnhoefer et al. [83]. Aβ42 was first dissolved in 20 mM sodium phosphate, 50 mM NaCl (pH 7.4) to prepare a 40 μM sample solution. The sample was then incubated quiescently at room temperature for 120 h. Finally, the Aβ42 sample was treated for 45 min in a cold ultrasonic bath.

Samples for ThT kinetics were prepared by introducing D3 and (or) Aβ42 seeds into freshly-dissolved Aβ42 in 20 mM sodium phosphate, 50 mM NaCl (pH 7.4). The concentration of Aβ42 monomer in all samples was 10 μM, and all samples contained 5 μM ThT. The concentrations of Aβ42 seeds were set to either 1% or 5% (*v*/*v*) (corresponding to 0.1 or 0.5 μM, respectively, based on monomer concentration) and the final concentration of D3 was 1 μM. A sample of 10 μM Aβ42 without seeds, but with 1 µM D3 was used as a control. Samples were pipetted to a 96-well plate, with each well containing 200 μL samples. The plate was then covered with a sealing film. ThT fluorescence of all samples was recorded using the same device and parameters, as described above for ThT assays. All samples were prepared in duplicate. Final data was normalized and averaged based on the repetitions.

### 4.7. Circular Dichroism Spectroscopy

Circular dichroism spectroscopy was applied to detect how D3 affects the secondary structure transition of Aβ42 under current experimental condition. Aβ42 aliquots were dissolved in 20 mM sodium phosphate, 50 mM NaF (pH 7.4—slightly alkali) to obtain a working concentration of 40 μM. NaF was used to substitute NaCl in this measurement to maintain the ionic strength of the buffer, so as to avoid the strong absorbance of NaCl in the far-UV area. For samples with D-peptide treatment, D3 was introduced into Aβ42 solutions to get a final concentration of 4 μM, equivalent to a molar ratio of 10:1 (Aβ42:D3). Besides, Aβ42 samples with D3 addition at 33 h were also included to test whether D3 is potent in pre-incubated Aβ42 samples. All samples were prepared on ice before being transferred to 1 mm path length quartz cuvettes. The final volume of each sample was 200 μL. Circular dichroism (CD) spectra were recorded from 260 nm to 190 nm using a J-815 spectropolarimeter (Jasco, Tokyo, Japan). All measurements were carried out with a step size of 0.5 nm and a bandwidth of 2 nm. The scanning speed was set to 100 nm/min and 10 scans were accumulated for each sample. Ellipticities at 198 nm and 217 nm were plotted against the incubation time to monitor the transition kinetics of Aβ42 samples in the absence or presence of D3. Samples were maintained at 20 °C within the measurement duration of 120 h, and were prepared in triplicate. CD spectra for all samples were deconvoluted using the online server Dichroweb [84,85] by applying the CDSSTR algorithm [86] and reference dataset Set 7 [87].

### 4.8. Turbidity Assay

Turbidity measurements were conducted by measuring the absorbance of the sample at 405 nm [88] to check whether D3 induces the formation of large Aβ42 aggregates in the time scale of the thermal equilibration in AUC experiments. Samples were prepared by dissolving Aβ42 aliquots in 20 mM sodium phosphate, 50 mM NaCl (pH 7.4) and adding D3 stock solution (2 mM). The final concentration of Aβ42 was set to 40 μM in all samples, and the molar ratios between Aβ42 and D3 were 10:1, 2:1, 1:1 and 1:2, respectively. Samples containing 40 μM Aβ42 alone were included as a control. 

The turbidity assay was carried out by measuring the spectra of all samples from 450 nm to 210 nm in a 1 cm quartz cuvette using a V-650 UV-Vis spectrophotometer (Jasco, Tokyo, Japan). The measurements were performed directly after preparing all samples, and repeated after 2 h of incubation at ambient temperature. All samples were subjected to a 15 min centrifugation at 726× *g* (equivalent to the speed during the calibration in AUC experiments) afterwards, and the supernatants were collected to measure the spectra again.

### 4.9. Atomic Force Microscopy

Atomic force microscopy imaging was performed to characterize morphologies of Aβ42 samples in the presence or absence of D3. In brief, 40 μM Aβ42 was incubated with or without 4 μM D3 in 20 mM sodium phosphate, 50 mM NaCl (pH 7.4—slightly alkali) at 20 °C. At 48 h and 120 h, 10 μL samples were pipetted onto freshly cleaved mica, and were further incubated at room temperature for 30 min. Mica with deposited samples were rinsed with ultrapure water for three times and finally dried with nitrogen gas. Atomic force microscopy (AFM) imaging was carried out in air at room temperature, using silicon cantilevers (OMCL AC160 TS, Olympus, Tokyo, Japan) and a JPK NanoWizard 3 microscope (JPK Instruments AG, Berlin, Germany) in AC mode. The nominal tip diameter of the cantilever was 7 nm. AFM height images at 10 × 10 μm^2^ and 1 × 1 μm^2^ (both with a resolution of 1024 × 1024 pixel) for all samples were taken with a line rate of 1 Hz and respective scanning speeds of 22.55 μm/s and 2.48 μm/s. Data was processed with JPK NanoWizard SPM data processing software.

## 5. Conclusions

Mechanistic insights into the mode of action of the compound D3 could be gained by strongly reducing the reactant concentrations. Submicromolar concentrations of Aβ42, which are close to the previously-determined critical concentration for the aggregation of 90 nM [89], as well as substoichiometric concentrations of D3, were applied in order to suppress the formation of precipitates. We conclude from our results that D3 is able to interact with Aβ42 monomers with submicromolar affinity, leading to the formation of complexes at 1:1 as well as other stoichiometries. The complexes are highly disordered and lack defined conformations. The addition of 0.1-fold D3 significantly slows down the fibrillation of Aβ42 by retaining Aβ in unstructured monomeric conformation, and thus by interfering with nuclei formation. Besides, D3 also slowed down the elongation of fibrillar structures. Our study demonstrates the versatile role of D3 in modulating the fibrillation of Aβ42 through targeting multiple events. The substoichiometric and diverse effects of D3 envision the promising application of its mode of action for the development of interventions in Alzheimer’s disease, but also in other protein misfolding-based pathologies.

## Figures and Tables

**Table 1:** Half completion time ($t_{1/2}$), slope (*k*) and lag time ($t_{lag}$) for fibrillation kinetics of 20 μM Aβ42 incubated with or without 2 μM D3. ^a^

Sample	$t_{1/2}(h)$	k	$t_{lag}(h)$
Aβ42	30.0 ± 0.7	0.15 ± 0.01	17.3 ± 1.6
Aβ42 + D3	79.3 ± 2.3	0.18 ± 0.07	67.6 ± 3.8

^a^ Data was obtained by fitting an empirical equation to thioflavin T (ThT) kinetics using AmyloFit [34].

## Supplementary Materials

The following are available online at https://www.mdpi.com/1420-3049/24/11/2129/s1, Table S1: Parameters used for evaluating sedimentation velocity data; Figure S1: MST analyses of fluorescein incubated with different concentrations of D3; Figure S2: MST analyses of the dissociation constant of FITC-Aβ42 and D3 in low ionic strength buffer; Figure S3: Initial fluorescence signals recorded for FITC-D3 in the presence of different concentrations of Aβ42 in analytical ultracentrifugation; Figure S4: Turbidity measurements of Aβ42 samples in the absence or presence of D3; Figure S5: Weight average s20,w of FITC-D3 and FITC-Aβ42 samples incubated with their binding partners by peak integration; Figure S6: Histograms of the s20,w values for the simulated Aβ42-D3 complexes in MD simulations; Figure S7: Analyses of ThT kinetics of Aβ42 in the absence or presence of substoichiometric D3 in sodium phosphate buffer; Figure S8: A comparison of the ThT kinetics of Aβ42 in the absence or presence of substoichiometric D3 in the sodium phosphate buffer and Tris-HCl buffer; Table S2: A summary of $t_{1/2}$, k and $t_{lag}$ for Aβ42 in the absence or presence of substoichiometric D3 in both buffers; Figure S9: The first scan of Aβ42, incubated with or without 0.1-fold D3 for 24 h acquired by the absorbance AUC; Figure S10: Sedimentation velocity analysis of Aβ42 incubated with or without 0.1-fold D3 for 24 h; Figure S11: CD spectra of D3 at two different concentrations; Figure S12: CD analyses of the effect of D3 addition (0.1-fold) after Aβ42 was incubated for 33 h.
