# Supplementary material for: Interference with Amyloid-β Nucleation by Transient Ligand Interaction
Source: Molecules. 2019 Jun 5;24(11):2129. doi: 10.3390/molecules24112129 (PMC6600523; doi:10.3390/molecules24112129)
Supplement: Supplementary file 1 [file molecules-24-02129-s001.pdf]

Supplementary Materials

Interference with amyloid β nucleation by transient ligand interaction

Tao Zhang[1,2], Jennifer Loschwitz[1,3], Birgit Strodel[1,3], Luitgard Nagel-Steger[1,2], Dieter Willbold[1,2*]

1 Institute of Complex Systems, Structural Biochemistry (ICS-6), Forschungszentrum Jülich, 52425 Jülich, Germany

2 Institut für Physikalische Biologie, Heinrich-Heine-Universität Düsseldorf, 40225 Düsseldorf, Germany

3 Institute of Theoretical and Computational Chemistry, Heinrich-Heine-Universität Düsseldorf, 40225 Düsseldorf, Germany

To whom the correspondence should be addressed:

d.willbold@fz-juelich.de

Table of Contents

Table S1	Parameters used for evaluating sedimentation velocity data	3
Figure S1	MST analyses of fluorescein incubated with different concentrations of D3	4
Figure S2	MST analyses of the dissociation constant of FITC-Aβ42 and D3 in low ionic strength buffer	4
Figure S3	Initial fluorescence signals recorded for FITC-D3 in the presence of different concentrations of Aβ42 in analytical ultracentrifugation	5
Figure S4	Turbidity measurements of Aβ42 samples in the absence or presence of D3	5
Figure S5	Weight average $s_{20,w}$ of FITC-D3 and FITC-Aβ42 samples incubated with their binding partners by peak integration	6
Figure S6	Histograms of $s_{20,w}$ values for the simulated Aβ42-D3 complexes in MD simulations	7
Figure S7	Analyses of ThT kinetics of Aβ42 in the absence or presence of substoichiometric D3 in sodium phosphate buffer	8
Figure S8	A comparison of ThT kinetics of Aβ42 in the absence or presence of substoichiometric D3 in sodium phosphate buffer and Tris-HCl buffer	8
Table S2	A summary of $t_{1/2}$, k and t_{lag} for Aβ42 in the absence or presence of substoichiometric D3 in both buffers	10
Figure S9	The first scan of Aβ42 incubated with or without 0.1-fold D3 for 24 h acquired by the absorbance AUC	9
Figure S10	Sedimentation velocity analysis of Aβ42, incubated with or without 0.1-fold D3 for 24 h Seeding kinetics of Aβ42 in the absence or presence of 0.1-fold D3	9
Figure S11	CD spectra of D3 at two different concentrations	10
Figure S12	CD analyses of the effect of D3 addition (0.1-fold) after Aβ42 was incubated for 33 h	10
References		11

Table S1. Parameters used for evaluating sedimentation velocity data at 20 °C.

Partial specific volume $\bar{\nu}$ (cm^3/g) [a]	FITC-Aβ42	0.732
	FITC-D3	0.667
Molecular mass (g/mol)	FITC-Aβ42	4974.6
	FITC-D3	2000
Buffer viscosity (P) [b]	20 mM sodium phosphate, 50 mM NaCl	0.01015
	55 mM Tris-HCl, 50 mM NaCl	0.01024
	H_2O	0.01002
Buffer density (g/cm^3) [b]	20 mM sodium phosphate, 50 mM NaCl	1.003
	55 mM Tris-HCl, 50 mM NaCl	1.002
	H_2O	0.9982

[a] Partial specific volumes of FITC-Aβ42 and FITC-D3 were calculated based on the amino acid composition and the formulated values for organic compounds documented by Durchschlag et al.[1].

[b] Buffer density and viscosity were calculated using Sednterp (version 20130813 BETA).

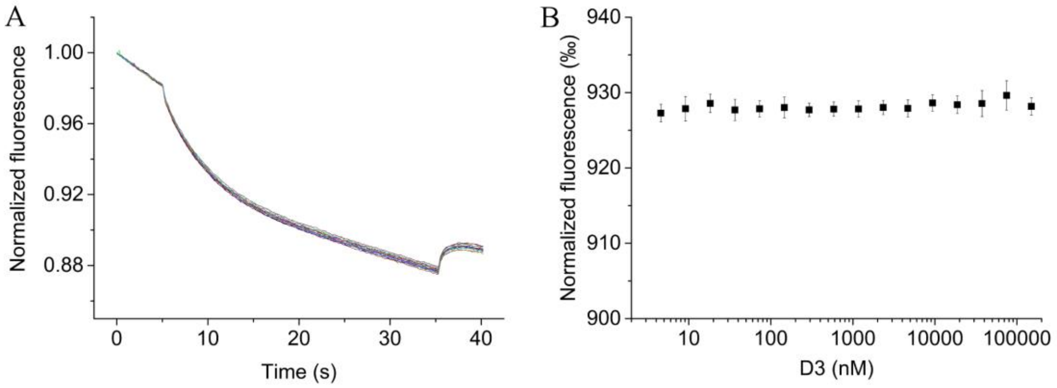

Figure S1. The fluorescein does not interact with D3. 40 nM fluorescein was titrated with different concentrations of D3 in 20 mM sodium phosphate, 50 mM NaCl (pH 7.4) at 22 °C. The time traces from one measurement was shown in A. The corresponding thermophoresis was shown in B. Samples were prepared in triplicate. The data was normalized and averaged.

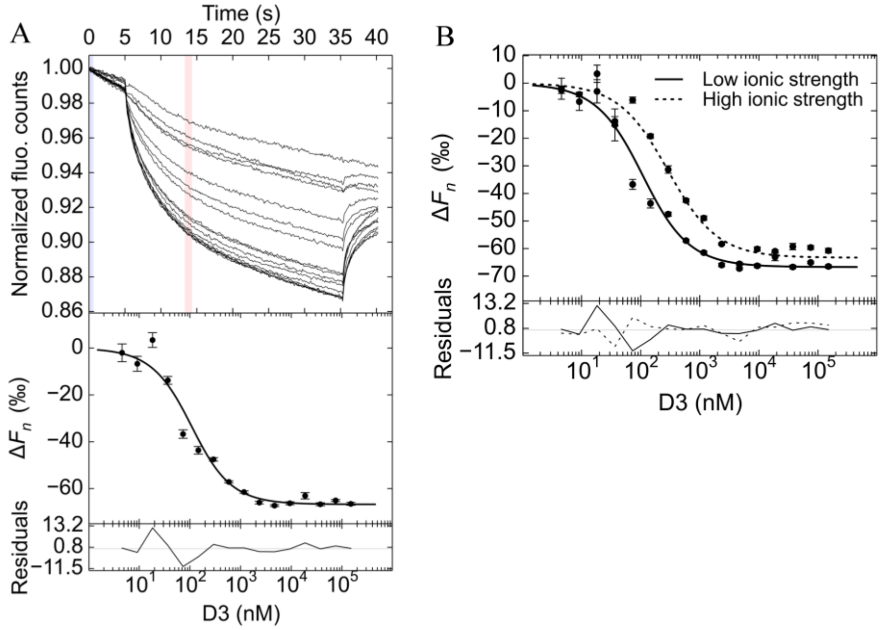

Figure S2. Determination of the dissociation constant of FITC-Aβ42 and D3 in low ionic strength buffer. 40 nM FITC-Aβ42 was incubated with different concentrations of D3 in 5 mM sodium phosphate, 50 mM NaCl (pH 7.4) in the presence of 0.01% (v/v) Tw20 at 22 °C. The experimental parameters for the measurement were the same as experiments shown in Figure 1 in the main text. The representative time traces and the fitting of the binding plot were shown in A. A comparison of the binding plots obtained in 5 mM sodium phosphate, 50 mM NaCl (low ionic strength, solid curve) and 20 mM sodium phosphate, 50 mM NaCl (high ionic strength, dotted curve) was shown in B.

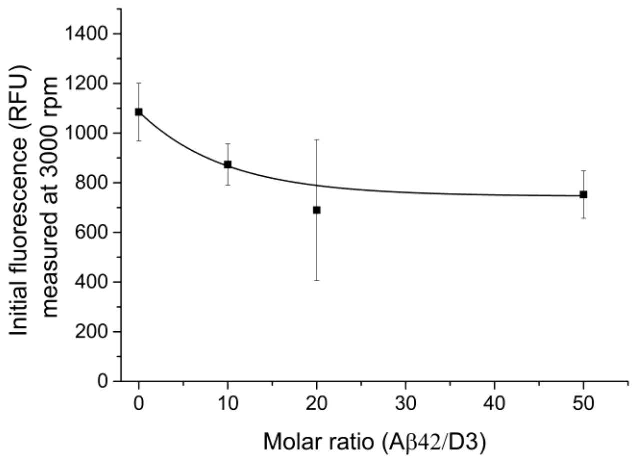

Figure S3. Initial fluorescence signals of 0.2 μM FITC-D3 incubated with different concentrations of freshly prepared Aβ42 in 20 mM sodium phosphate, 50 mM NaCl, 0.0004% (w/v) PEI. 100 μl samples were loaded into 3-mm double sector titanium cells and were incubated for 2 h prior to the centrifugation for thermal equilibration. Fluorescence signals were recorded using the fluorescence detection system while the centrifuge was spinning at 3000 rpm (726 *g*). Data was averaged based on five independent experiments.

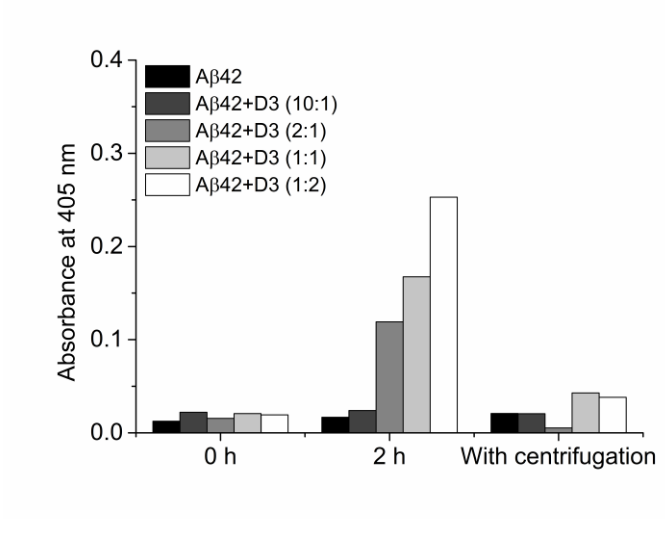

Figure S4. Turbidity measurements of Aβ42 in the absence of presence of D3. 40 μM Aβ42 was incubated with 4, 20, 40 and 80 μM D3 in 20 mM sodium phosphate, 50 mM NaCl, 0.01% Tw20 (pH 7.4) at ambient temperature. The turbidity expressed by the absorbance at 405 nm was determined by scanning the spectrum of the sample using a UV-Vis spectrophotometer. The centrifugation was performed using a benchtop centrifuge at 726 *g* for 15 min.

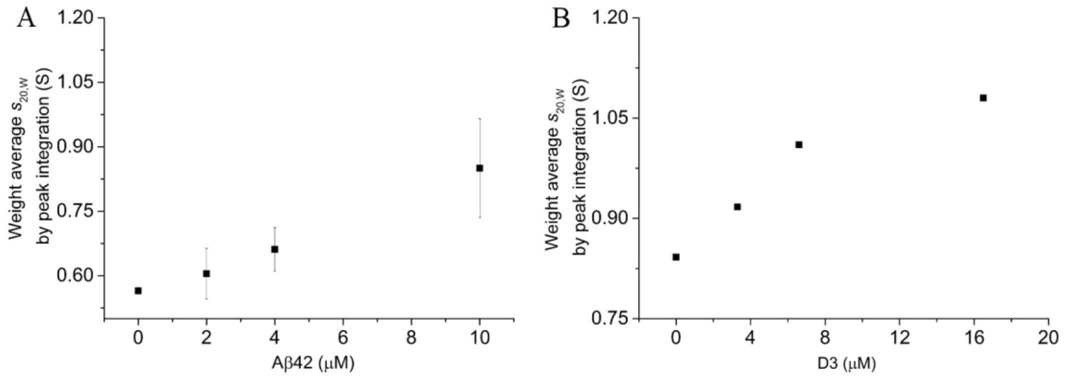

Figure S5. Weight average $s_{20,w}$ of FITC-D3 incubated with Aβ42 (A) and FITC-Aβ42 incubated with D3 (B) based on the peak integration of *s*-value distributions obtained from *c*(*s*) analyses shown in Figure 2 in the main text.

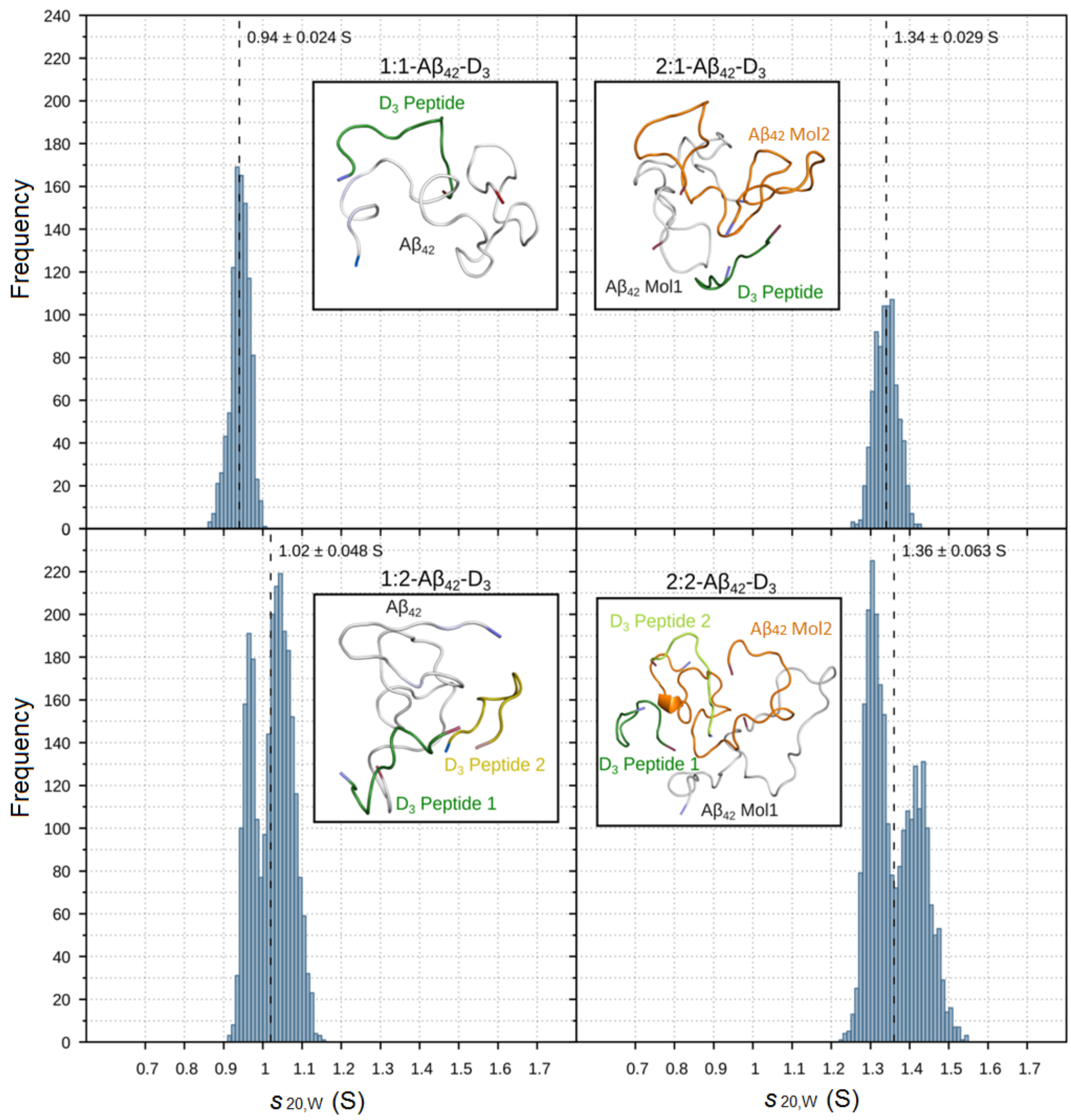

Figure S6. Histograms of the $s_{20,w}$ values for the simulated Aβ42-D3 complexes. The representative conformation taken from the most populated cluster of each complex stoichiometry is shown as an insert. The average $s_{20,w}$ value with standard deviation is given for each stoichiometry.

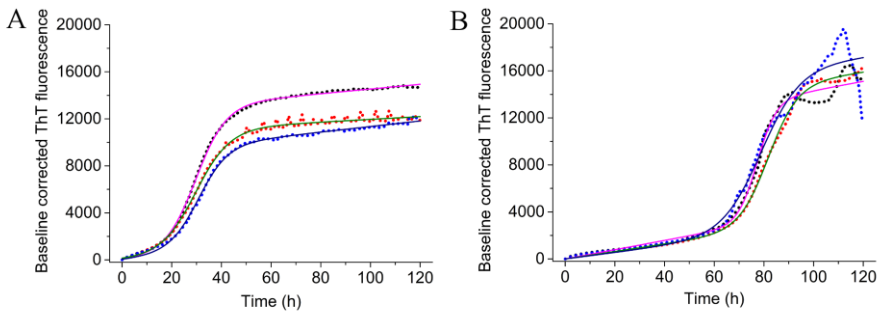

Figure S7. Analyses of ThT kinetics of 20 μM Aβ42 in the absence (A) or in the presence (B) of 2 μM D3 in 20 mM sodium phosphate, 50 mM NaCl, pH 7.4 at 20 °C using AmyloFit and the equation (1) as described in the materials and methods. ThT data was processed in AmyloFit to correct the baseline. Raw data after processing is expressed in dot curves, and fitting is shown as solid curves. Samples were prepared in triplicate.

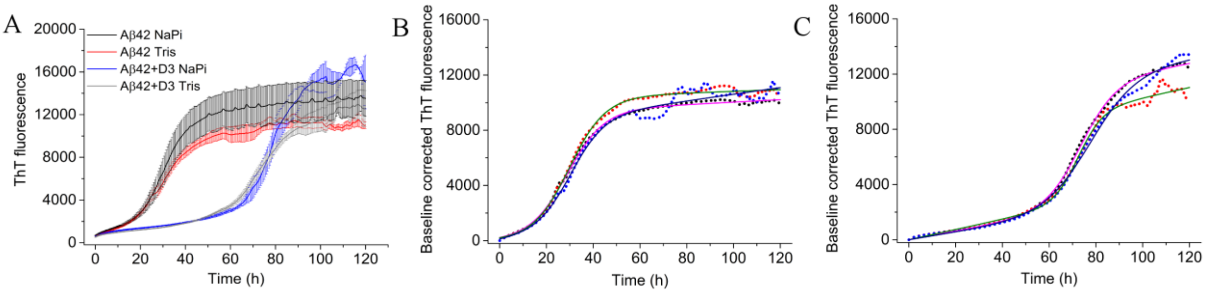

Figure S8. Comparison of ThT kinetics of 20 μM Aβ42 incubated with or without 2 μM D3 in 20 mM sodium phosphate, 50 mM NaCl (pH 7.4) or 55 mM Tris-HCl, 50 mM NaCl (pH 7.4) (A). AmyloFit analyses of ThT kinetics of 20 μM Aβ42 alone (B) and 20 μM Aβ42 with 0.1-fold D3 (C) are shown. The data was processed in AmyloFit to correct the baseline. Raw data after processing is expressed in dot curves, and fitting is shown as solid curves. Samples were prepared in triplicate.

Table S2. AmyloFit analyses of ThT kinetics of Aβ42 incubated with or without 0.1-fold D3 in sodium phosphate buffer or Tris-HCl buffer at 20 °C.[a]

	Buffer	$t_{1/2}$ (h)	k	t_{lag} (h)
Aβ42	sodium phosphate	30.0 ± 0.7	0.15 ± 0.01	17.3 ± 1.6
Aβ42	Tris-HCl	29.7 ± 0.8	0.13 ± 0.01	14.5 ± 1.4
+D3 (10:1)	sodium phosphate	79.3 ± 2.3	0.18 ± 0.07	67.6 ± 3.8
+D3 (10:1)	Tris-HCl	74.5 ± 2.8	0.14 ± 0.04	59.3 ± 2.2

[a] Data was obtained by fitting the kinetics with equations [2,3] shown in the materials and methods section using the online webserver AmyloFit [4].

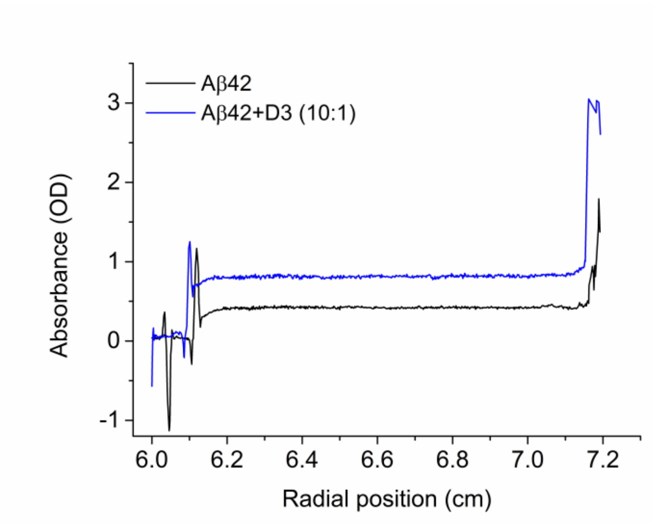

Figure S9. The first scan of the sedimentation profiles of 20 μM Aβ42 incubated without or with 2 μM D3 measured at 210 nm, 45,000 rpm by the absorbance based AUC.

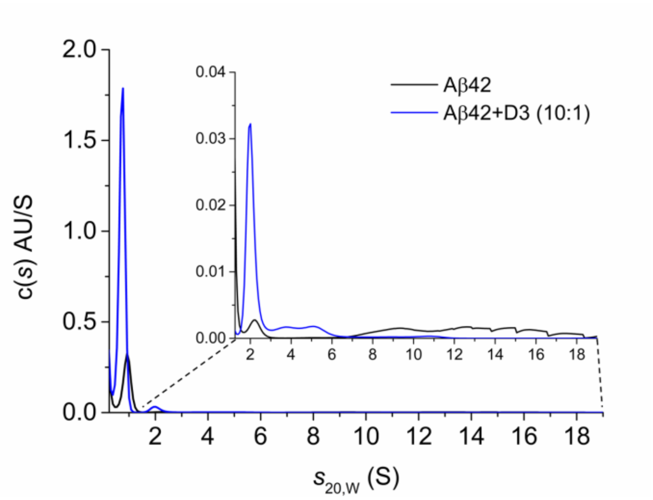

Figure S10. Sedimentation velocity analysis of Aβ42 incubated with or without 0.1-fold D3 for 24 h. Aβ42 at 20 μM was incubated without or with 2 μM D3 in 20 mM sodium phosphate, 50 mM NaCl (pH 7.4) at 20 °C for 24 h. Samples were analyzed in an AUC with absorbance detection at 45,000 rpm for 15.5 h and sedimentation profiles were evaluated using $c(s)$ analysis to obtain $s_{20,w}$ values. The insert shows the distribution between 1.25 and 19 S.

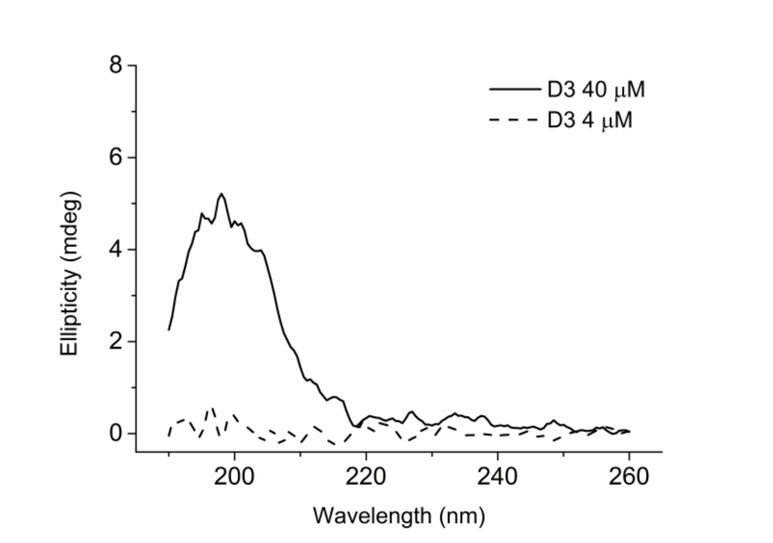

Figure S11. CD spectra of 4 (dashed) and 40 μM (solid) D3 in 20 mM sodium phosphate, 50 mM NaF (pH 7.4). The positive peak at 198 nm represents the mirror image of the random coil structure for a conventional L-enantiomeric peptide.

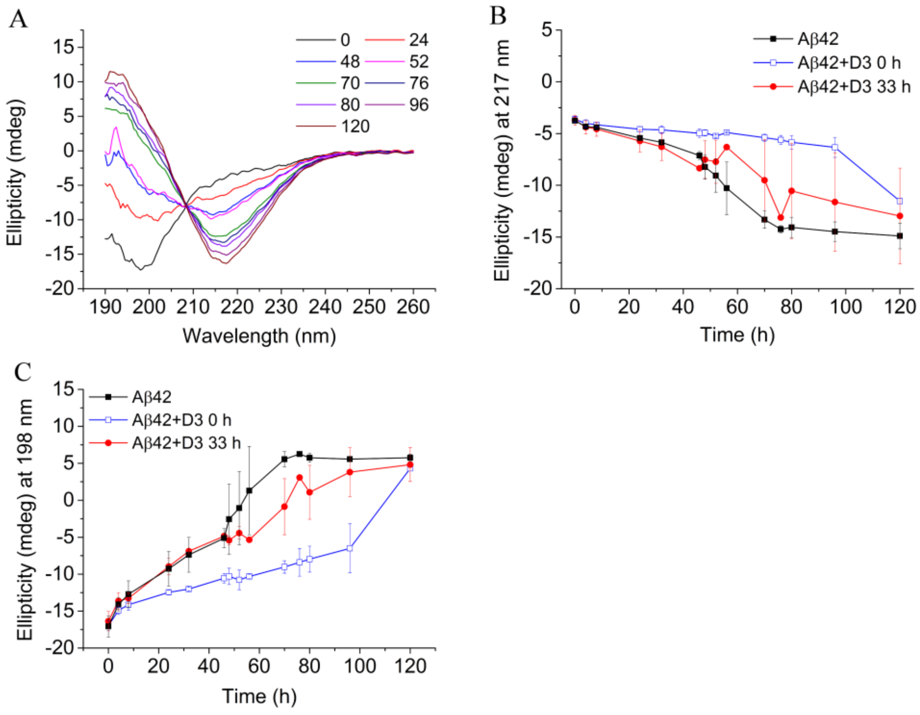

Figure S12. CD analyses showing the effect of delayed addition of 0.1-fold D3 on the secondary structure transition of Aβ42. 4 μM D3 was added after 40 μM Aβ42 was incubated for 33 h in 20 mM sodium phosphate, 50 mM NaF (pH 7.4) at 20 °C. The CD spectra at selected time points (h) were shown in A. The conversion kinetics by plotting ellipticities at 217 nm (B) or 198 nm (C) against the incubation time were compared with Aβ42 alone and Aβ42 with D3 addition from the beginning. Data was averaged based on triplicate.

References

1. Durchschlag , H.; Zipper, P. Calculation of the partial volume of organic compounds and polymers. In *Progress in Colloid & Polymer Science* Springer: 1994; Vol. 94, pp. 20-39.
2. Hellstrand, E.; Boland, B.; Walsh, D.M.; Linse, S. Amyloid beta-protein aggregation produces highly reproducible kinetic data and occurs by a two-phase process. *ACS chemical neuroscience* **2010**, *1*, 13-18, doi:10.1021/cn900015v.
3. Luo, J.; Yu, C.H.; Yu, H.; Borstnar, R.; Kamerlin, S.C.; Graslund, A.; Abrahams, J.P.; Warmlander, S.K. Cellular polyamines promote amyloid-beta (Abeta) peptide fibrillation and modulate the aggregation pathways. *ACS chemical neuroscience* **2013**, *4*, 454-462, doi:10.1021/cn300170x.
4. Meisl, G.; Kirkegaard, J.B.; Arosio, P.; Michaels, T.C.; Vendruscolo, M.; Dobson, C.M.; Linse, S.; Knowles, T.P. Molecular mechanisms of protein aggregation from global fitting of kinetic models. *Nature protocols* **2016**, *11*, 252-272, doi:10.1038/nprot.2016.010.
